# The global dynamic transmissibility of COVID-19 and its influencing factors: an analysis of control measures from 176 countries

**DOI:** 10.1186/s12889-023-15174-0

**Published:** 2023-02-28

**Authors:** Hongjian Wang, Yajia Lan

**Affiliations:** West China School of Public Health and West China Fourth Hospital, Sichuan University, Chengdu, China

**Keywords:** COVID-19, Transmissibility, *R*_0_, Control measures, Dynamic bayesian network

## Abstract

**Objective:**

To summarise the dynamic characteristics of COVID-19 transmissibility; To analyse and quantify the effect of control measures on controlling the transmissibility of COVID-19; To predict and compare the effectiveness of different control measures.

**Methods:**

We used the basic reproduction number (${R}_{0}$) to measure the transmissibility of COVID-19, the transmissibility of COVID-19 and control measures of 176 countries and regions from January 1, 2020 to May 14, 2022 were included in the study. The dynamic characteristics of COVID-19 transmissibility were summarised through descriptive research and a Dynamic Bayesian Network (DBN) model was constructed to quantify the effect of control measures on controlling the transmissibility of COVID-19.

**Results:**

The results show that the spatial transmissibility of COVID-19 is high in Asia, Europe and Africa, the temporal transmissibility of COVID-19 increases with the epidemic of Beta and Omicron strains. Dynamic Bayesian Network (DBN) model shows that the transmissibility of COVID-19 is negatively correlated with control measures. Restricting population mobility has the strongest effect, nucleic acid testing (NAT) has a strong effect, and vaccination has the weakest effect.

**Conclusion:**

Strict control measures are essential for controlling the COVID-19 outbreak; Restricting population mobility and nucleic acid testing (NAT) have significant impacts on controlling the COVID-19 transmissibility, while vaccination has no significant impact. In light of these findings, future control measures may include the widespread use of new NAT technology and the promotion of booster immunization.

## Introduction

The novel coronavirus pneumonia (NCP) caused by COVID-19 is a highly infectious disease with the widest global transmission in human history. With the emergence of variants of concern (VOC) such as Alpha, Beta, Gamma, Delta, and Omicron strains, COVID-19 has exhibited a trend of decreased virulence [1] but increasing transmissibility [2]. As of August 31, 2022, data from WHO shows that 600,443,074 people have been infected and 6,485,216 have died worldwide. In light of these figures, controlling COVID-19 transmission remains a critical public health issue.

To curb the ongoing spread of COVID-19, three major control measures have been implemented worldwide: Nucleic acid testing (NAT), vaccination and restrictions on population mobility. Their implementation had a significant impact on social and economic activities [3]. Analysing the dynamic transmissibility of COVID-19 and quantifying the effectiveness of these measures can assist in striking a balance between global public health and economic activities. This is essential in minimizing the negative impact of COVID-19 pandemic.

Since the onset of the COVID-19 pandemic, there has been a shortage of research with a global perspective to quantify the impact of multiple control measures. In 2020, researchers first described the transmissibility of COVID-19 using basic reproduction number (${R}_{0}$) [4–6], and initially clarified the effectiveness of restrictions on population mobility [3]. In 2021, following the widespread implementation of nucleic acid testing and vaccination, single-factor studies demonstrated that nucleic acid testing restricted COVID-19 transmissibility [6], while vaccines had limited effectiveness due to immune escape [7]. In 2022, researchers focused on the dynamic transmissibility of variants of concern, and have begun to quantify the effectiveness of multiple control measures in specific areas [8]. Due to limitations in localized data sources or the singularity of control measures, the lack of generalizability makes it challenging to compare the effectiveness of different COVID-19 control measures.

In our research, we first present an overview of COVID-19 dynamic transmissibility and spatio-temporal characteristics. We then construct a Dynamic Bayesian Network (DBN) to quantify the effectiveness of three major COVID-19 control measures and compare scenarios under strict and relaxed control measures. The innovations of our research are threefold: Firstly, our research covers a broad time horizon of the pandemic, from January 2020 to August 2022, and a global spatial horizon of 176 countries and territories, which removes the temporal and spatial limitations of previous analyses; Secondly, we quantify the effectiveness of three control measures by incorporating them into a single formula, which allows for a direct comparison of each control measure and combinations of control measures; Lastly, our research can be utilized to predict the dynamics transmissibility of COVID-19 control measures, which can serve as a valuable reference for predicting COVID-19 outbreaks and for deploying medical resources globally. The findings of our research are generalisable and provide a global reference for the evaluation of control measures for COVID-19, which will aid in promoting global health equity and minimizing the negative impact of COVID-19 on economic globalisation.

## Methodology

### Data

Our data source is from the project of the Global Change Data Lab called ‘Our World in Data’ [9]. Weekly mean basic reproduction number (${R}_{0}$), weekly mean nucleic acid testing (NAT) rate, vaccination rate and weekly mean stringency index of COVID-19 from January 1, 2020 to May 14, 2022 in 176 countries and regions are used as variables. Since the variables are monotonically increasing, we employ Hermite interpolation to interpolate any missing data.

### Dynamic characteristics of COVID-19 transmissibility

The basic reproduction number (${R}_{0}$) is a key indicator for understanding the transmissibility of infectious diseases. When ${R}_{0}$<1, the spread of COVID-19 is expected to decrease, and vice versa. As such, it can be used to measure the effectiveness of control measures.

To explore the spatial characteristics, we calculated maximum weekly ${R}_{0}$ of COVID-19 by continent and visualised the ${R}_{0}$ of COVID-19 for each district using a graduated colour map. To explore the temporal characteristics, we graphically illustrated the weekly ${R}_{0}$ of COVID-19 and used the Savitzky-Golay filter to extract the trend of transmissibility. Additionally, we also identified the date when variants of concern were found to investigate any possible correlation.

### Quantification of the effect of COVID-19 control measures

**Fig. 1:**
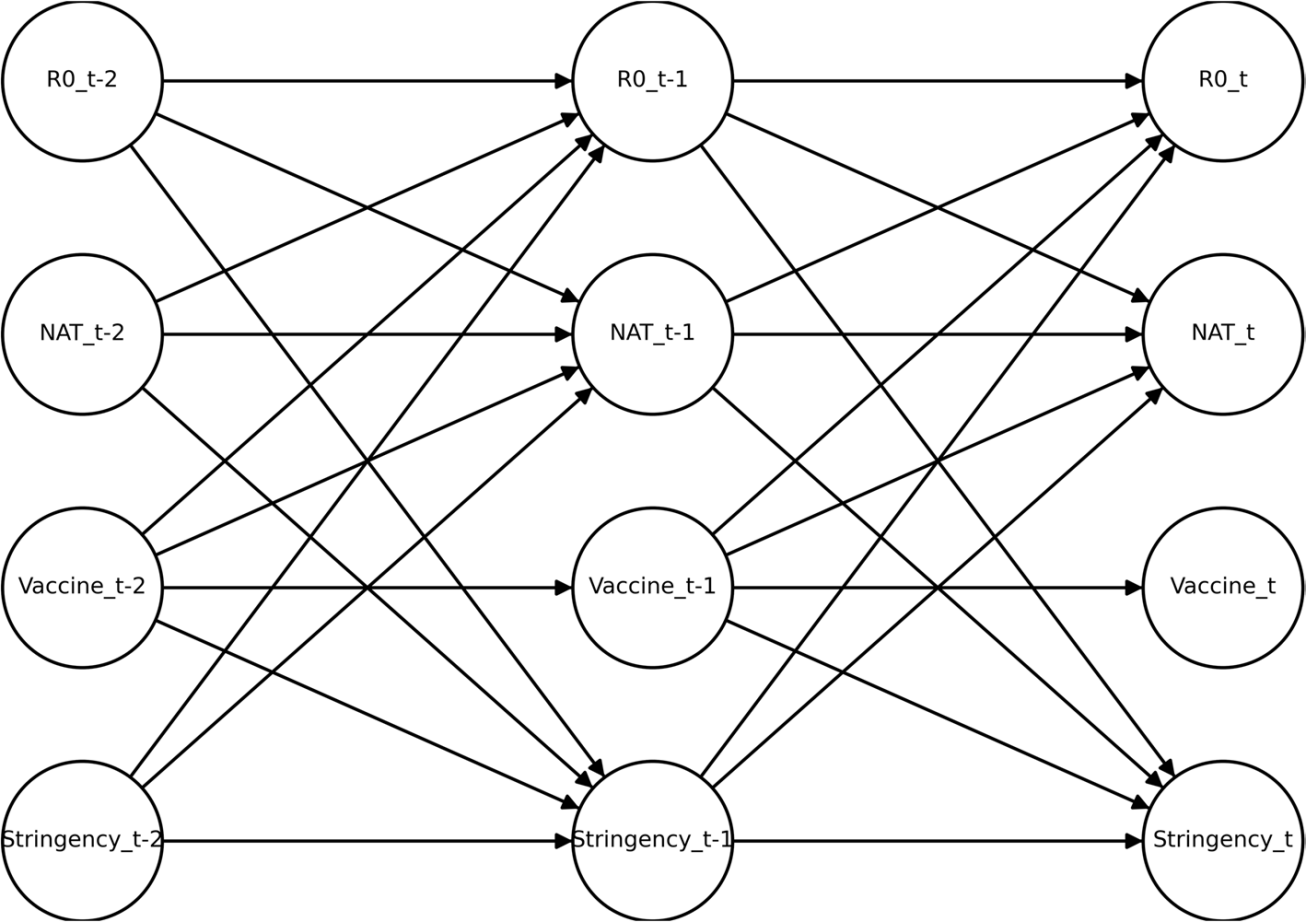
DBN structure assumption

The PCMCI algorithm is a statistical method that can estimate causality with time lags and control for false positives [10]. In this case, the algorithm was used to analyse the correlation between control measures and the transmissibility of COVID-19. The incubation period of COVID-19 ranges from 3 to 7 days [11], which was taken into account by using a lag of one week in the algorithm, and a significance level of 0.05 was used. Edges pointing to vaccination were removed because vaccination is a planned event. These details are shown in Fig. 1.

With the correlations we also constructed a DBN (Dynamic Bayesian Network) model using Bayesian linear regression to quantify the effect of control measures. The basic reproduction number (${R}_{0}$) should satisfy the multiplicative relationship with the independent variables of the previous time slice due to its strong autocorrelation.

$$\frac{{R}_{t}+{\alpha }_{1}}{{R}_{t-1}+ {\alpha }_{2}}= {\beta }_{1}{X}_{NAT}^{t-1}+{\beta }_{2}{X}_{vaccine}^{t-1}+{\beta }_{3}{X}_{stringency}^{t-1}+{\alpha }_{3}$$

The formula can also be expressed in the following form:

$${R}_{t}= \left({R}_{t-1}+{\alpha }_{2}\right)\left({\beta }_{1}{X}_{NAT}^{t-1}+{\beta }_{2}{X}_{vaccine}^{t-1}+{\beta }_{3}{X}_{stringency}^{t-1}+{\alpha }_{3}\right)-{\alpha }_{1}$$

It is assumed that the basic reproduction number (${R}_{0}$) and the variables of the previous time slice satisfy the above relations, where ${R}_{t}$ is the basic reproduction number of COVID-19 at time t, ${R}_{t-1}$ is the basic reproduction number of COVID-19 at time t-1. ${\beta }_{1}$,${\beta }_{2}$ and ${\beta }_{3}$ are the effect coefficients of nucleic acid testing, vaccination and the stringency index. ${\alpha }_{1}$, ${\alpha }_{2}$ and ${\alpha }_{3}$ are constant terms.

### Evaluation of COVID-19 control measures effect

With the DBN model, we compared the strictest control measures from Asia to the loosest control measures from Africa. We visualised the effect of differences between these options and the downtrend of COVID-19 transmissibility.

### Statistical software

The python 3.9.7 software was used for statistical analysis. The matplotlib and geopandas packages were utilized to plot the dynamic spatio-temporal transmission of COVID-19. The tigramite package was utilized for structure learning of the Dynamic Bayesian Network (DBN), and the pymc3 package was used for parameter learning of the DBN.

## Result

### Dynamic transmissibility of COVID-19

#### Spatial transmissibility of COVID-19

**Fig. 2:**
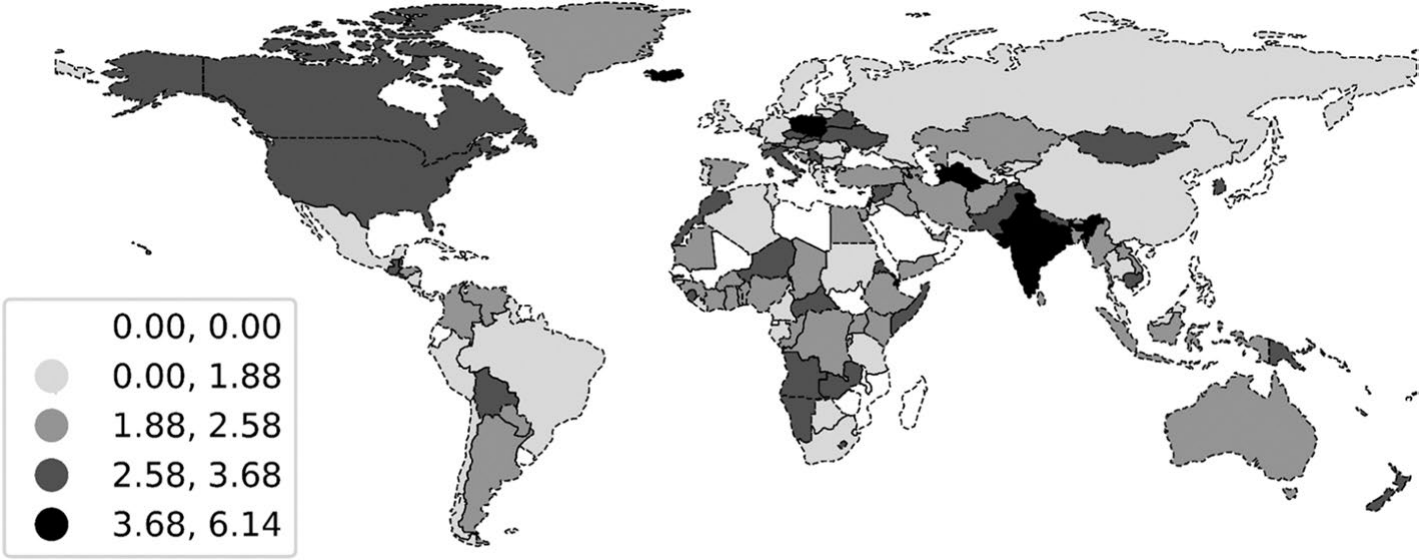
Spatial transmissibility of COVID-19 by country

Classified by continent, result shows that Asia has the highest transmissibility with a value of 1.836, indicating that the outbreak in Asia is not effectively controlled and should be taken seriously; Europe has the second largest mean value, but the fourth largest maximum value of ${R}_{0}$, indicating that the transmissibility in Europe is severe but less volatile; The mean value of ${R}_{0}$ in Africa is close to that in Europe, but the maximum value is higher in Africa, indicating that transmissibility in Africa is more prone to outbreaks; The mean ${R}_{0}$ in the Americas is more average, and the overall epidemic severity in South America and North America is similar; Oceania has the lowest mean ${R}_{0}$ value, which may be related to the lower population density and population mobility in Oceania [12, 13]. These details are shown in Table 1 and Fig. 2.

**Table 1:** Spatial transmissibility of COVID-19 by continent

Continent	Mean	SD	Max
Asia	1.836	0.704	6.138
Europe	1.559	0.597	3.559
Africa	1.531	0.635	4.498
North America	1.369	0.546	3.655
South America	1.248	0.490	3.017
Oceania	1.191	0.534	2.517
World	1.456	0.417	3.238

#### Temporal transmissibility of COVID-19

**Fig. 3:**
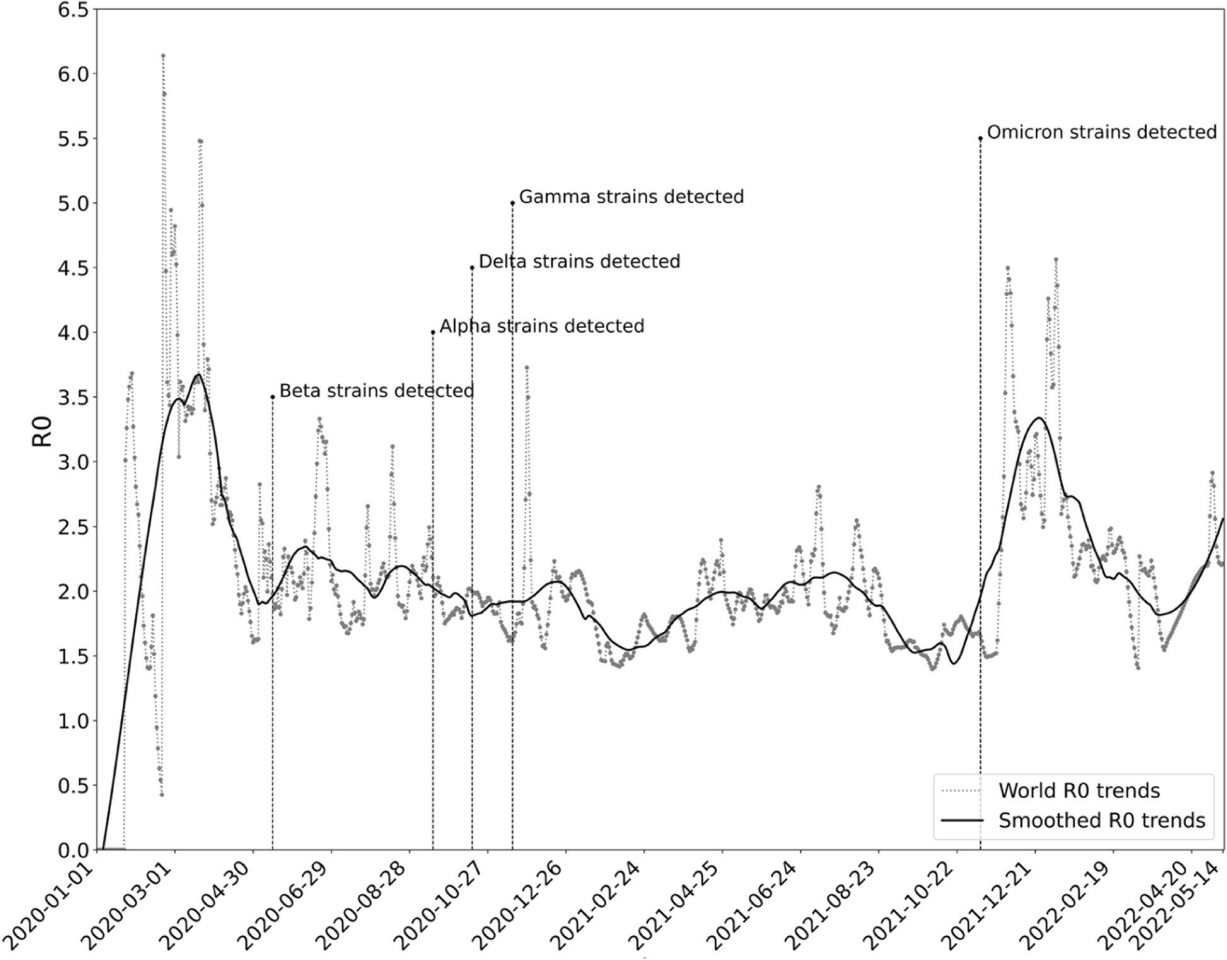
Temporal transmissibility of COVID-19

**Fig. 4:**
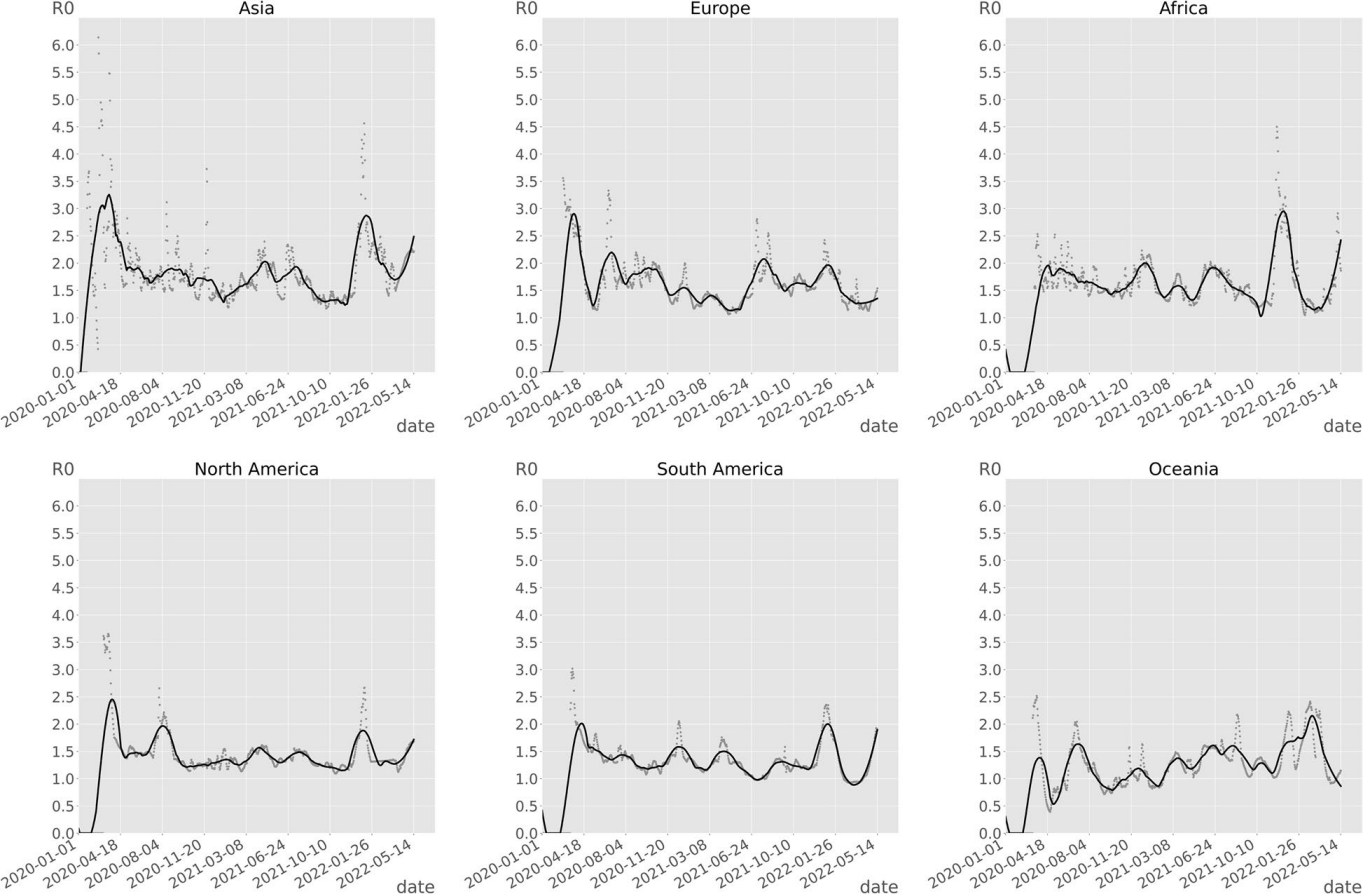
Temporal transmissibility of COVID-19 by continent

The emergence of variants of concern (VOC) is closely linked to the fluctuations of the COVID-19 epidemic [14]. According to Figs. 3 and 4, it can be seen that the main prevalent strains of COVID-19 from the beginning to April 2021 was the original strain. Asia was considered to be the first region where COVID-19 was detected, with transmissibility reaching a peak of 6.1383 on February 21, 2020; from April to October 2021, the main prevalent strains were the Alpha strain prevalent in Europe and North America [15], the Gamma strain prevalent in South America [16], and the Delta strain prevalent in Asia [17]. From October 2021 to May 2022, the major epidemic strain is the Omicron strains, which was endemic worldwide [18]. The spatial transmissibility of COVID-19 increased with the epidemic of Beta and Omicron strains, and remained steady with the epidemic of Alpha, Delta, and Gamma strains.

### Quantification of the effect of COVID-19 control measures

#### Dynamic Bayesian Network structure learning

**Fig. 5:**
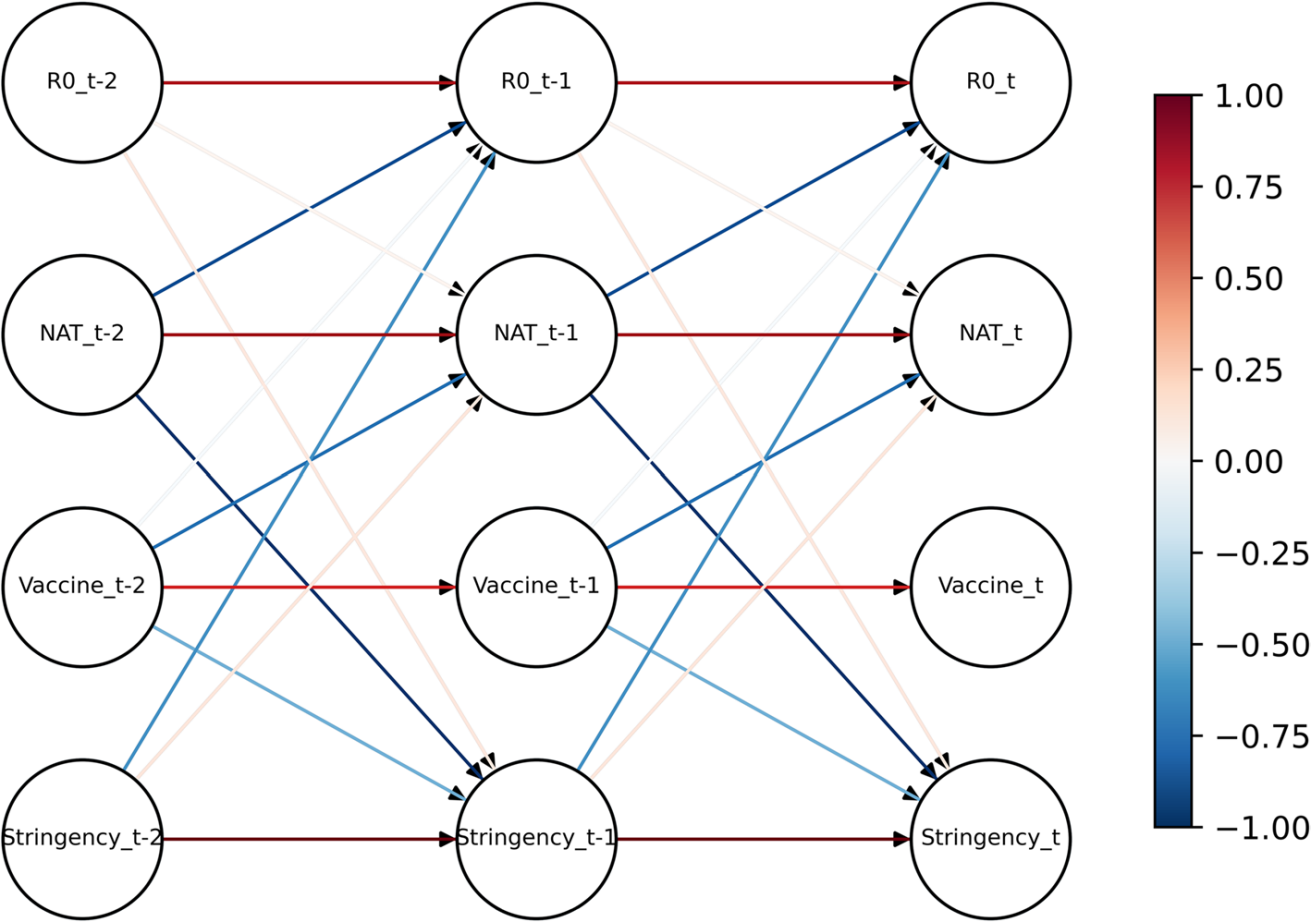
DBN structure of COVID-19 transmissibility

The PCMCI algorithm shows that nucleic acid testing, vaccination and the stringency index are all negatively correlated with the basic reproduction number with a lag of one week. This suggests that control measures may help control the spread of the epidemic [19]. The causal inference results of the specific correlations are shown in Fig. 5.

#### Dynamic Bayesian Network parameter learning

**Fig. 6:**
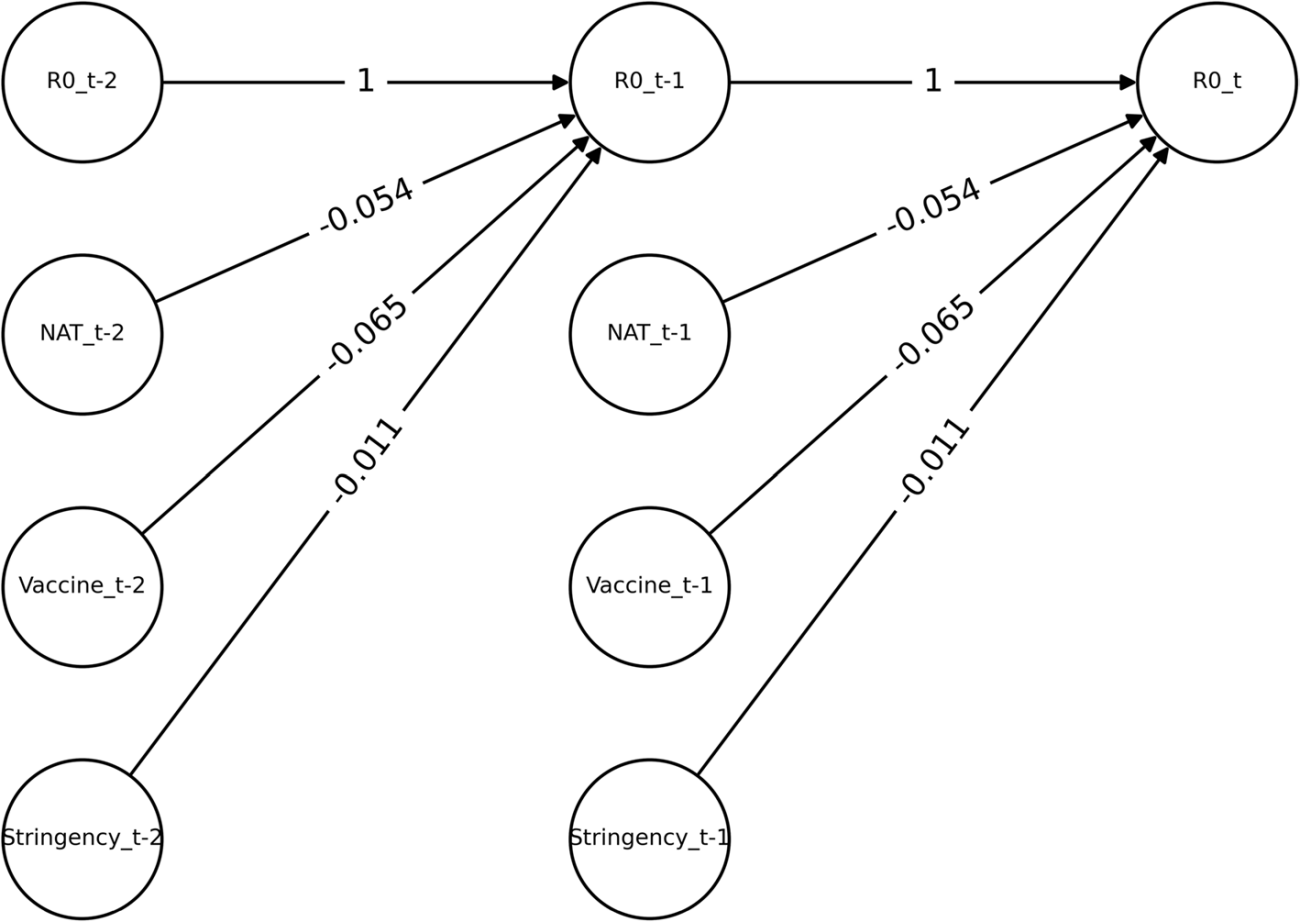
DBN of COVID-19 transmissibility

In this study, we used the first 500 Markov chain Monte Carlo samples as a prior distribution and solved for a posterior distribution using 1500 samples. The regression results are shown in Table 2 and Fig. 6. The Markov chain standard errors (MSCE) are all less than 0.001, the Gelman-Rubin statistic statistics of the model are all less than 1.1, indicating that the model converges well. The coefficient of determination R2 was 0.863, which means that the independent variables explained the dependent variable well in the regression analysis.

**Table 2:** DBN parameters of COVID-19 transmissibility

	Mean	SD	3%HDI	97%HDI
${\beta }_{1}$	-0.054	0.002	-0.058	-0.051
${\beta }_{2}$	-0.011	0.001	-0.014	-0.008
${\beta }_{3}$	-0.065	0.002	-0.069	-0.061
${\alpha }_{1}$	-0.038	< 0.001	0.038	0.038
${\alpha }_{2}$	0.009	0.001	0.009	0.010
${\alpha }_{3}$	0.998	0.002	0.995	1.001

The coefficients ${\beta }_{1}$, ${\beta }_{2}$ and ${\beta }_{3}$ are all less than 0, indicating a negative correlation between nucleic acid testing, vaccination and stringency index with ${R}_{0}$. Vaccination (${\beta }_{2}$=-0.011) has the weakest inhibitory effect on ${R}_{0}$, while nucleic acid testing (${\beta }_{1}$=-0.054) has a more obvious inhibitory effect and stringency index (${\beta }_{3}$ = -0.065) has the strongest inhibitory effect on ${R}_{0}$.

### Evaluation of COVID-19 control measures effect

According to the DBN model, the basic reproduction number (${R}_{0}$) follows the formula:

$$\frac{{R}_{t}-0.038}{{R}_{t-1}+0.009}=-0.054*{X}_{NAT}^{t-1}-0.011*{X}_{vaccine}^{t-1}-0.065*{X}_{stringency}^{t-1}+0.998$$

The formula can also be expressed in the form:

$${R}_{t}= \left({R}_{t-1}+0.009\right)\left(-0.054*{X}_{NAT}^{t-1}-0.011*{X}_{vaccine}^{t-1}-0.065*{X}_{stringency}^{t-1}+0.998\right)+0.038$$

**Fig. 7:**
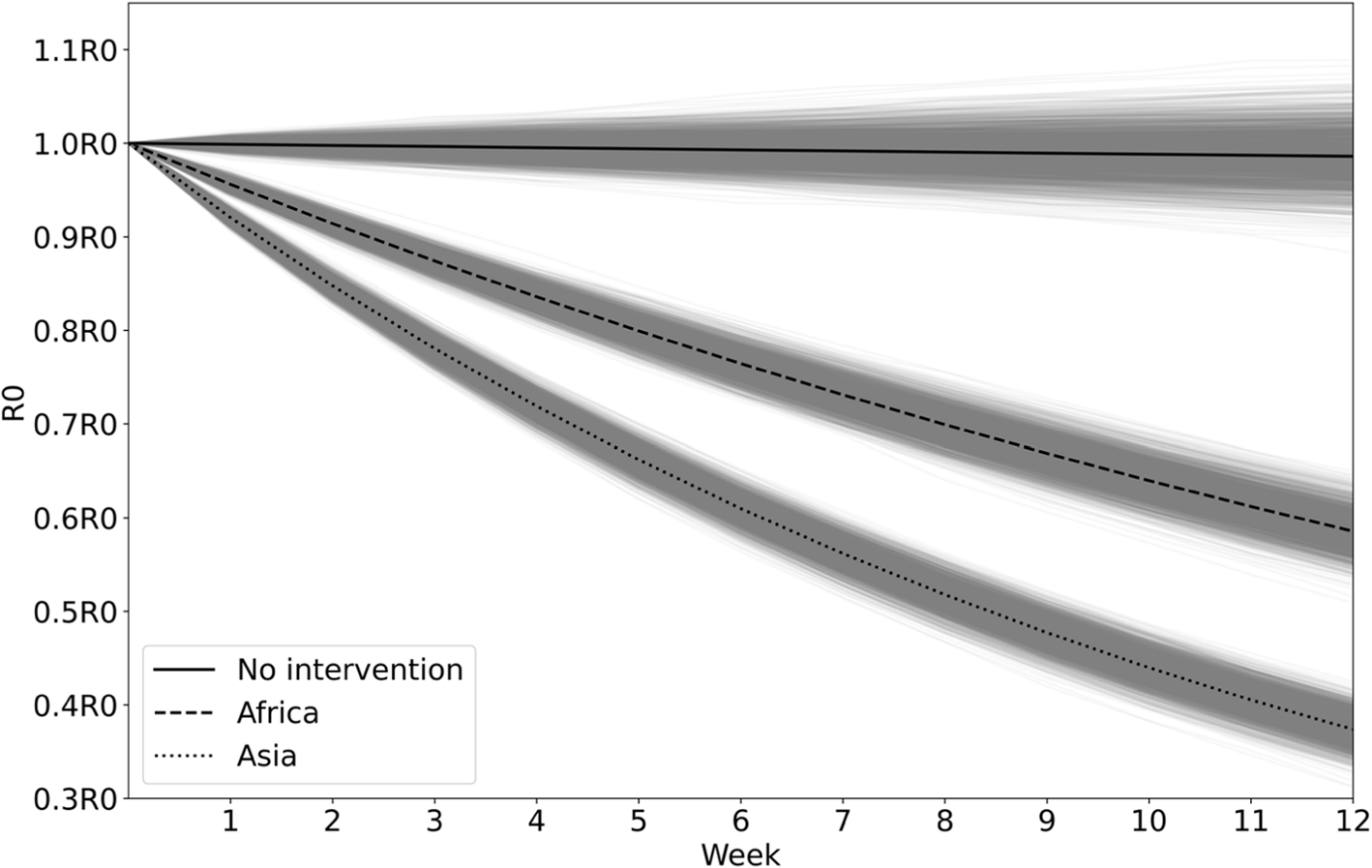
Transmissibility under different control measures

According to Table 3, Asia had the strongest control measures interventions while Africa has the weakest interventions. The control measures of Asia and Africa were brought into the model for one million Monte Carlo simulations and the results are shown in Fig. 7, with darker shaded areas indicating higher probability.

**Table 3:** COVID-19 control measures by continent

Continent	NAT		Vaccination rate		Stringency index	
	normalization	%	normalization	%	normalization	%
Asia	0.133	13.286	0.502	50.172	0.719	71.901
Europe	0.113	11.261	0.533	53.274	0.654	65.356
North America	0.056	5.602	0.455	45.455	0.707	70.732
South America	0.044	4.366	0.567	56.738	0.655	65.514
Oceania	0.034	3.415	0.440	44.034	0.668	66.808
Africa	0.023	2.286	0.134	13.415	0.642	64.216

Strict control measures can reduce COVID-19 transmissibility by half within 12 weeks. The worldwide average value of ${R}_{0}$ is 1.456, and with Asian controls measures, COVID-19 can be effectively controlled within 6 weeks. If less strict control measures are implemented or none at all, the transmissibility will remain high or even increase slightly. Stricter control measures can accelerate the containment of epidemics of similar severity.

## Discussion

### Strict control and nucleic acid testing can reduce transmissibility

Our research found that stringency index has a significant impact on reducing the transmissibility of COVID-19 within a week [19]. Even though the value of coefficient (${\beta }_{3}$=-0.065) is small, strict control measures have an exponential effect and, significantly accelerate the decrease of ${R}_{0}$ to less than 1, thereby controlling the spread of the virus. Additionally, our research found that nucleic acid testing has a significant impact of on reducing the transmissibility COVID-19 within a week (${\beta }_{1}$=-0.054). This is because nucleic acid testing allows for early diagnosis, detection, isolation and treatment of COVID-19 cases [20], which has a significantly suppresses the development of outbreaks.

Therefore, to slow the spread of COVID-19, it is practical to close schools [21], workplaces [22] and public transportation [23] for a period of time, to screen and restrict public events and public gatherings [24], and to control large population movements such as travel and business trips [25]. In addition, extensive nucleic acid testing can aid in controlling outbreaks, and various control measures should be implemented based on available resources.

### COVID-19 vaccination cannot reduce transmissibility

Vaccination is a prerequisite for forming an immune barrier if the vaccination rate reaches a threshold and the virus does not have significant immune escape [26]. However, COVID-19 is an RNA virus which is prone to mutation, and Omicron strain has been identified with high transmissibility and significant immune escape [27]. The development of COVID-19 vaccines fails to keep pace with the emergence of new viral strains. Research has shown that while vaccination can help reduce the rate of severe illnesses and deaths, it may not be as effective in preventing transmission [28]. Therefore, it is not advisable to rely solely on vaccination to ease restrictions on controlling highly transmissible and mutable respiratory diseases.

### Future outlook

For countries with populations and high population density [29], if no control measures are implemented at all, the spread of COVID-19 may lead a run on medical resources; if excessive control measures are implemented, it can harm economic growth and waste medical resources [30]. A more reasonable approach is seeking a balance between global public health and economic activities [31]. Our findings are practical in predicting epidemics and deploying resources,, which determine the time of application of control measures based on the current transmission of COVID-19, quantify and predicts the effect of control measures. Our findings also predict the future trend of the transmission of COVID-19 based on the current control measures to prevent excessive liberalization leading to a resurgence of the epidemic.

### Future outlook

The limitations of nucleic acid testing include long waiting times and high labour costs for manual testing. Faster and decentralised nucleic acid testing technology has emerged and has the potential to be implemented on a larger scale. If nucleic acid testing technology further matures, then large-scale, high-frequency nucleic acid testing will help control the epidemic [32], reduce the need for strict control measures, and accelerate the recovery of social and economic activities.

Another approach is to increase vaccination booster rates, further reduce the rate of severe illness and mortality [33]. Additionally, it is important to ensure that the healthcare system has the capacity to handle critically ill patients and that medical resources are sufficient for both public health and the efficient operation of clinical systems.

### Limitations of the study

The limitation of this study is that the data is country- and region-specific, lacking country- and region-specific provincial (continental) and municipal data. Further studies should improve the accuracy of the study by using higher precision data, in combination with GIS systems, to quantify the impact of control measures on the transmission of COVID-19.

## Conclusion

The dynamic temporal transmissibility of COVID-19 is still high in Asia, Europe, and Africa, but low in North America, South America, and Oceania. The spatial transmissibility of COVID-19 increases with the epidemic of Beta and Omicron strains. In these circumstances, controlling the transmission of COVID-19 remains a critical public health issue.

Dynamic Bayesian Network (DBN) model shows that strict control measures are essential for controlling COVID-19 outbreaks, which can reduce COVID-19 transmissibility by half within 12 weeks. Restricting population mobility and nucleic acid testing have significant impacts on controlling COVID-19 transmissibility, while vaccination has no significant impact. Future control measures for COVID-19 may involve widespread use of new nucleic acid testing technology and increased access to booster immunizations.

It is important for countries and regions to understand the effectiveness of these measures and strive for a balance between public health and economic development to promote global health equity. Both overly strict and overly lenient control measures are not advisable, it is necessary to comprehensively consider medical resources and economic conditions, to improve the utilization efficiency of medical resources as much as possible, and to maintain global health equity to the greatest extent.

## Data Availability

The data is publicly available. The datasets generated and analysed during the current study are available in the Oxford COVID-19 Government Response Tracker repository, https://ourworldindata.org/coronavirus.
